# Tapered high-gain Fabry–Perot cavity antenna with high sidelobe suppression for 5G industry

**DOI:** 10.1038/s41598-023-42716-8

**Published:** 2023-09-21

**Authors:** Muhammad Hussain, Kyung-Geun Lee, Dongho Kim

**Affiliations:** 1 Network Research Lab (NRL), Department of Information and Communication Engineering, Sejong University, Seoul, 05006 Republic of Korea; 2 Antenna and RF Applications Lab (ARFAL), Department of Electrical Engineering, Sejong University, Seoul, 05006 Republic of Korea

**Keywords:** Electrical and electronic engineering, Electronics, photonics and device physics

## Abstract

We propose a Fabry–Perot cavity (FPC) antenna to suppress a sidelobe level (SLL) while maintaining a reasonably high gain. Generally, conventional FPC antennas (FPCAs) produce a high SLL because waves in their FPC leak considerably through lateral openings, which is a primary reason for lowering antenna gains. We propose two design approaches to solve this problem: the reflection magnitude tapering of a partially reflective surface (PRS) and considering different incident modes for the PRS design. First, the proposed tapering can remarkably reduce an SLL by providing the PRS with more radiation opportunities. Second, the different incident modes of transverse electric (TE) and transverse magnetic (TM) can increase an antenna gain by considering a more realistic illumination environment of the PRS. We have proven that our antenna provides 19.8 dBi realized gain with high sidelobe suppression (SLS) of more than 23 dB. Consequently, the proposed FPCA can suppress sidelobes significantly while maintaining a high gain. Good agreement between simulations and experiments demonstrates the usefulness of our proposal.

## Introduction

Conventional array antennas have been found in many practical applications^1–7^ due to their directional radiation patterns and versatile beamforming ability. Generally, these antennas have massive and complex feed structures^8–10^, which induce unwanted power loss. Moreover, they also suffer from high sidelobes, which is an important index for many applications such as the 5G industry^11–14^, urban air mobility (UAM), radar systems^15^, and wireless power transfer^16,17^.

Transmitarrays^18^, reflectarrays^19,20^, and resonance cavity antennas^21,22^ have been widely explored to overcome these problems, which also include power pattern reconfigurability^20,21^. However, high sidelobes are still an obstacle for many applications.

To improve the gain and sidelobe suppression (SLS) properties of FPCAs, several PRS superstrates—a single-layer PRS^23,24^, an inhomogeneous gradient-index PRS^25^, and a multilayered PRS with different permittivity^26^, a phased gradient surface^27^, a phase correction structure^28^, a double-layer nonuniform superstrate^29^, an FPC with side walls^30^—have been proposed. However, for the required properties, the PRSs are too complex to be engineered with a standard PCB process; even with them, the SLS performance is still not satisfactory.

To solve these problems, we propose a novel FPCA with a tapered PRS to maximally suppress side lobes with a reasonably high antenna gain. The tapered PRS provides gradually decreasing reflection magnitudes, which helps reduce undesirable energy leakage through lateral openings.

We newly introduce artificial magnetic conductors (AMC) cells to a reflectarray ground plane to minimize the expected gain reduction, which is designed to enforce Trentini’s resonance condition in the cavity with corresponding tapered PRS cells^31^. All simulations were conducted using CST Studio Suite^32^.

## Design and operation

**Figure 1:**
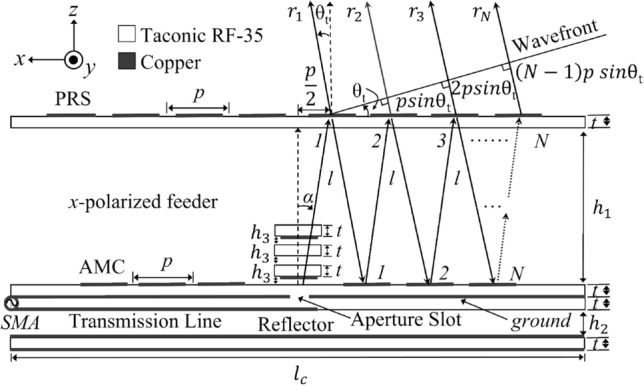
Operational principle of the proposed FPCA with $\alpha = \tan^{ - 1} \left( {p/2h_{1}^{ } } \right) = 26.6^\circ$, *l* = *h*_1_*/cosα* = 33.54 mm, *h*_1_ = 0.52 *λ*_0_ = 31.6 mm, *h*_2_ = 10 mm, *h*_3_ = 0.5 mm, *λ*_0_ = 60 mm (at *f*_0_ = 5 GHz) , *l*_*c*_ = 4.33 *λ*_0_ = 260 mm, *p* = 0.5 *λ*_0_ = 30 mm, *t* = 1.52 mm, θ_t_ = 0° (broadside direction), *N* = 4 for PRS unit cells and *N* = 3 for AMC unit cells.

The design model of the proposed FPCA is depicted in Fig. 1, which consists of a tapered PRS superstrate, a reflectarray, and an *x*-polarized aperture-coupled stacked microstrip patch antenna at the center of the FPCA. Here, a transmission line runs under the reflectarray to transfer electromagnetic energy to the stack patches through the slot. The reflectarray and transmission line substrates are firmly bonded by a 0.04 mm epoxy FR4 layer with relative permittivity (*ε*_*r*_) = 4.3 and loss tangent (tan *δ*) = 0.025^33^. A 1.52 mm thick Taconic RF-35 substrate is used to fabricate the FPCA, which has *ε*_*r*_ = 3.5 and tan δ = 0.0025. Additionally, to minimize unwanted back radiation (in the–*z*-direction)^34^, we intentionally install the reflector at optimum height *h*_2_, which also largely affects the impedance matching of our antenna^35^.

### Operation of the FPCA

The operational principle of the proposed FPCA is depicted in Fig. 1, providing a constructively built wavefront toward the target direction (θ_t_). As well known, to obtain a high gain in the broadside direction, all the rays need to satisfy Trentini’s resonance condition^31^, which is written as

1 $$\phi_{\Gamma n}^{PRS} - 2\beta h_{1}^{{}} - \pi = \pm 2N\pi$$

where *β* = 2*π/λ*_0_ is the free space phase constant, *λ*_0_ is a wavelength in free space at design frequency (*f*_0_) 5 GHz, and *h*_1_ is the cavity height. $\phi_{\Gamma n}^{PRS}$ is the reflection phase of the uniform PRS. *N* and *n* are the total and the *n*^th^ number of unit cells, respectively.

Conventional FPCAs consist of a uniform PRS and a metallic ground plane. Hence, we do not need to consider the transmission behavior of the PRS to derive FP resonance because all the PRS cells provide the same transmission phase. That is why we do not include the transmission property in (1).

However, when a PRS is tapered as ours, each cell exhibits different reflection and transmission properties, which does not satisfy the required FP resonance condition with a fixed cavity height. In addition, as we know, it is pretty tricky to control the reflection and transmission phase of the PRS unit cell independently. It is even more challenging with a single-layer PRS providing only limited reflection phase coverage.

To overcome this problem, we devise a reflectarray ground plane consisting of AMC unit cells to provide a desirable wide-enough reflection phase range. Consequently, we can form a highly-directive main beam satisfying the FP resonance condition with the help of the AMC cells. For the PRS and the AMC cell distribution in Fig. 1, following the ray from the source gives corresponding phase delays written as

2 $$\varphi_{1} = - \beta l + \phi_{T1}^{PRS}$$

3 $$\varphi_{2} = - 3\beta l + \phi_{\Gamma 1}^{PRS} + \phi_{\Gamma 1}^{AMC} + \phi_{T2}^{PRS} - \beta p\sin \theta_{t}$$

4 $$\varphi_{3} = - 5\beta l + \phi_{\Gamma 1}^{PRS} + \phi_{\Gamma 1}^{AMC} + \phi_{\Gamma 2}^{PRS} + \phi_{\Gamma 2}^{AMC} + \phi_{T3}^{PRS} - 2\beta p\sin \theta_{t}$$

5 $$\varphi_{n} = - (2n - 1)\beta l + \phi_{\Gamma 1}^{PRS} + \phi_{\Gamma 1}^{AMC} + \phi_{\Gamma 2}^{PRS} + \phi_{\Gamma 2}^{AMC} + ... + \phi_{Tn}^{PRS} - (n - 1)\beta p\sin \theta_{t}$$

Here, *φ*_*n*_ is the phase delay of the *n*th ray *r*_*n*_ in Fig. 1 on the wavefront. All the phase delays (*φ*_1_, *φ*_2_, *φ*_3_, …, *φ*_*n*_) should be equal on the wavefront to produce intensive radiation at the target angle θ_t_. Its rigorous derivation is provided in the Supplementary Section 1 "The rigorous derivation of the overall phase delay within the cavity". Comparing each phase delay from (2) to (5) yields

6 $$\,\phi_{\Gamma n}^{AMC} = 2\beta l - \phi_{\Gamma n}^{PRS} + \phi_{Tn}^{PRS} - \phi_{Tn + 1}^{PRS} + \beta p\sin \theta_{t}$$

where *l* = *h*_1_*/cosα*. The $\phi_{\Gamma n}^{PRS}$ and $\phi_{Tn}^{PRS}$ are the reflection and transmission phases of the *n*th PRS cell, and $\phi_{\Gamma n}^{AMC}$ is the reflection phase of the *n*th AMC cell. Therefore, we can confirm that introducing AMC cells makes it easier to enforce constructive interference in any target direction.

### Reflection magnitude tapering of the PRS superstrate

**Figure 2:**
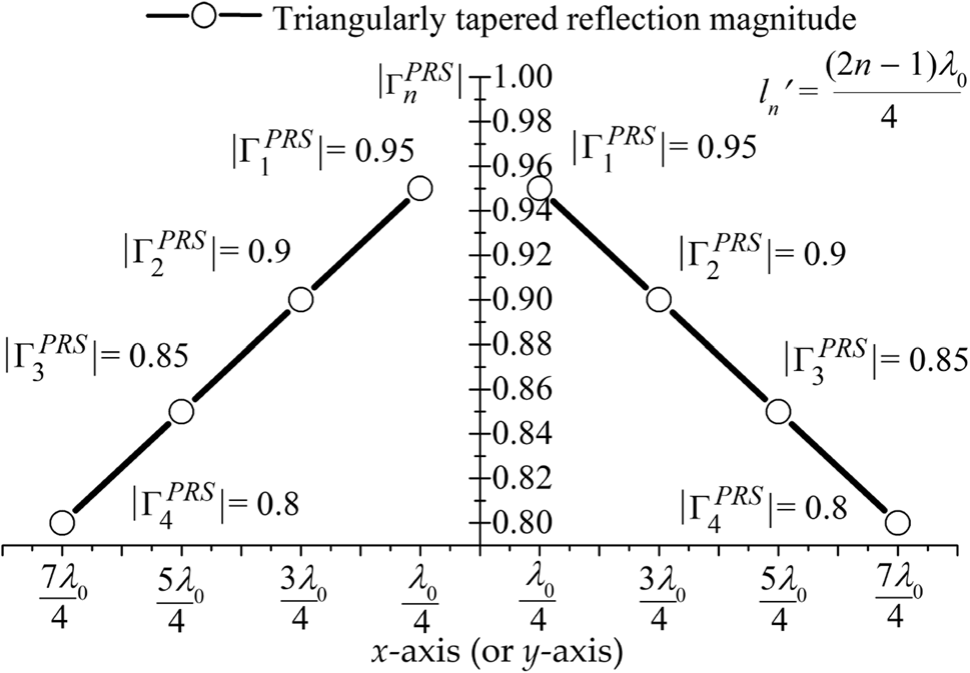
Triangular tapering applied to the reflection magnitude of the PRS.

The remarkably enhanced SLS is one of the essential goals of this work. So, we apply triangular tapering providing both high SLS and gain to the reflection magnitude of PRS cells ^36^, which is shown in Fig. 2 and written as

7 $$\left| {\Gamma_{n}^{PRS} } \right| = \left| {\Gamma_{\min }^{PRS} } \right| + \left| {\Gamma_{OTR}^{PRS} } \right|\left( {1 - \frac{2}{{l_{c} }}\left| {l_{n}^{\prime } } \right|} \right)$$

Here, $|\Gamma_{n}^{PRS} |$ is the reflection magnitude of the *n*th PRS unit cell, $l_{n}{\prime} = \left( {2n - 1} \right)\lambda_{0} /4$, and $l_{c}^{ } = 4.3 \lambda_{0}$ is a physical aperture length of the FPCA. $\left| {\Gamma_{OTR}^{PRS} } \right| = \left| {\Gamma_{max}^{PRS} } \right| - \left| {\Gamma_{min}^{PRS} } \right|$ is the tapered reflection magnitude range of the PRS. After intensive simulations, we tapered the reflection magnitude of the PRS cell to $0.8 \le \left| {\Gamma_{n}^{PRS} } \right| \le 0.95$.

**Figure 3:**
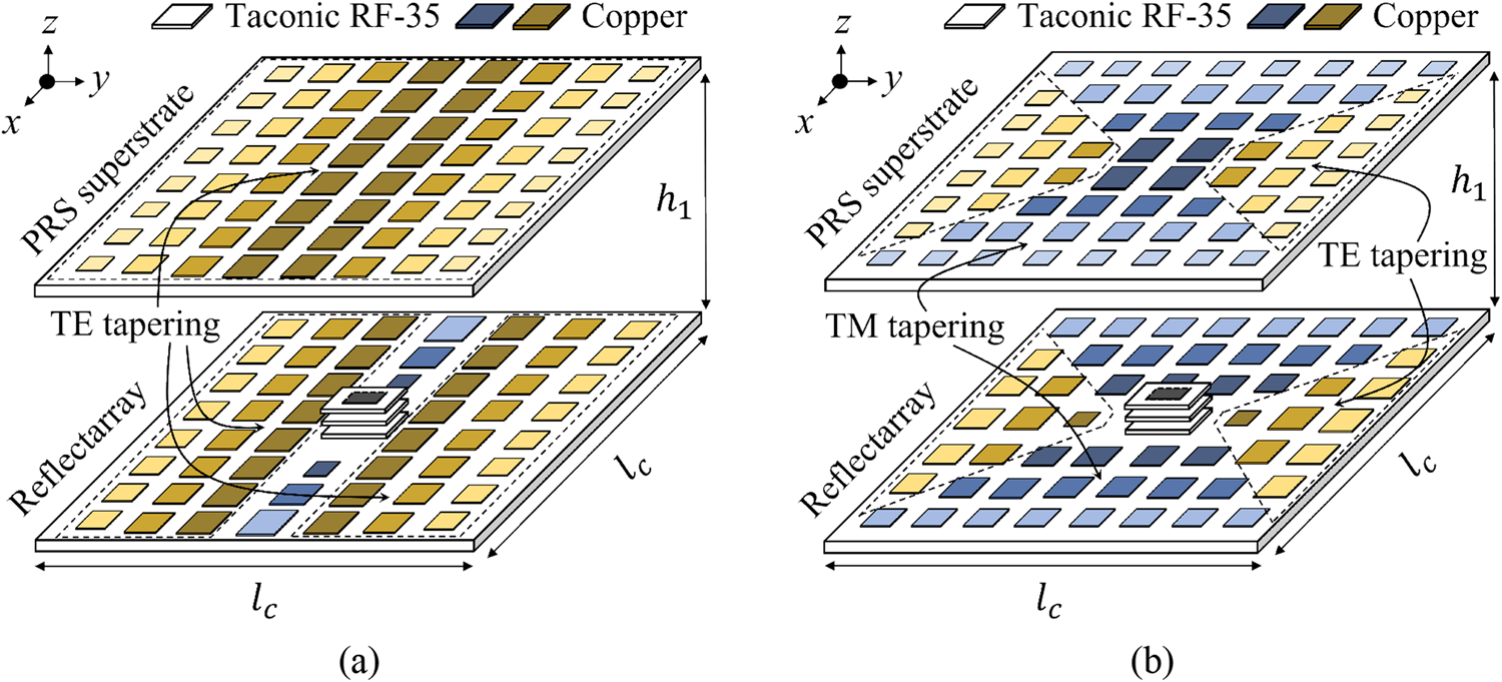
The FPCA models illustrate the proposed (**a**) 1-D TE tapering and (**b**) 2-D full TE-TM tapering.

The overall geometry of the proposed FPCA is illustrated in Fig. 3. Here, we will intentionally try three different tapering methods to analyze the effect of them: TE tapering (Fig. 3a), TM tapering [90° rotated version of Fig. 3a except the feeder], and full TE-TM tapering (Fig. 3b).

**Figure 4:**
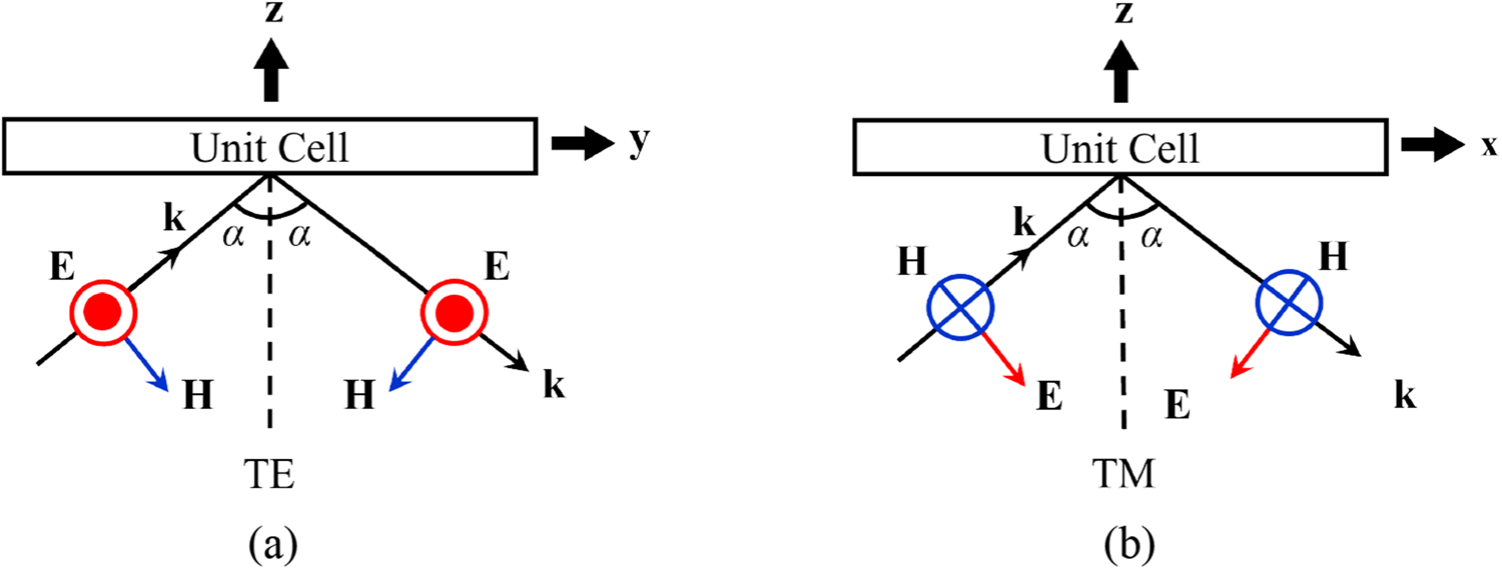
Two distinct incidence modes used for PRS and AMC unit cell simulations: (**a**) a TE mode and, (**b**) a TM mode with an incidence angle *α.*

Considering that we use the *x*-polarized source feeder, incidence along the *x*- and *y*-axis becomes TM and TE modes, respectively, as shown in Fig. 4. Here, the incidence angle ($\alpha ={\mathit{tan}}^{-1}\left(p/{2h}_{1}\right)$, in Fig. 1) is fixed at 26.6°.

In Fig. 3a, we expect highly suppressed sidelobes along the *y*-axis because we applied the TE tapering along the axis (*ϕ* = 90°). The deployment of the unit cells is described in detail in Supplementary Section 2 "PRS and AMC unit cells deployment for 1-D TE or TM tapering". Similarly, we can readily change Fig. 3a to TM tapering just by rotating the whole structure by 90° except for the feeding part.

Until now, we have demonstrated one-dimensional (1-D) tapering, i.e., TE or TM tapering along only one axis. However, it is worth trying tapering along both the *x*- and *y*-axes simultaneously for higher SLS, which is named 2-D (full TE-TM) tapering hereafter. In Fig. 3b, the 2-D tapering is applied to the PRS according to TE and TM incidences (see Fig. 4).

### Design of the feeder, the PRS, and the reflectarray

**Figure 5:**
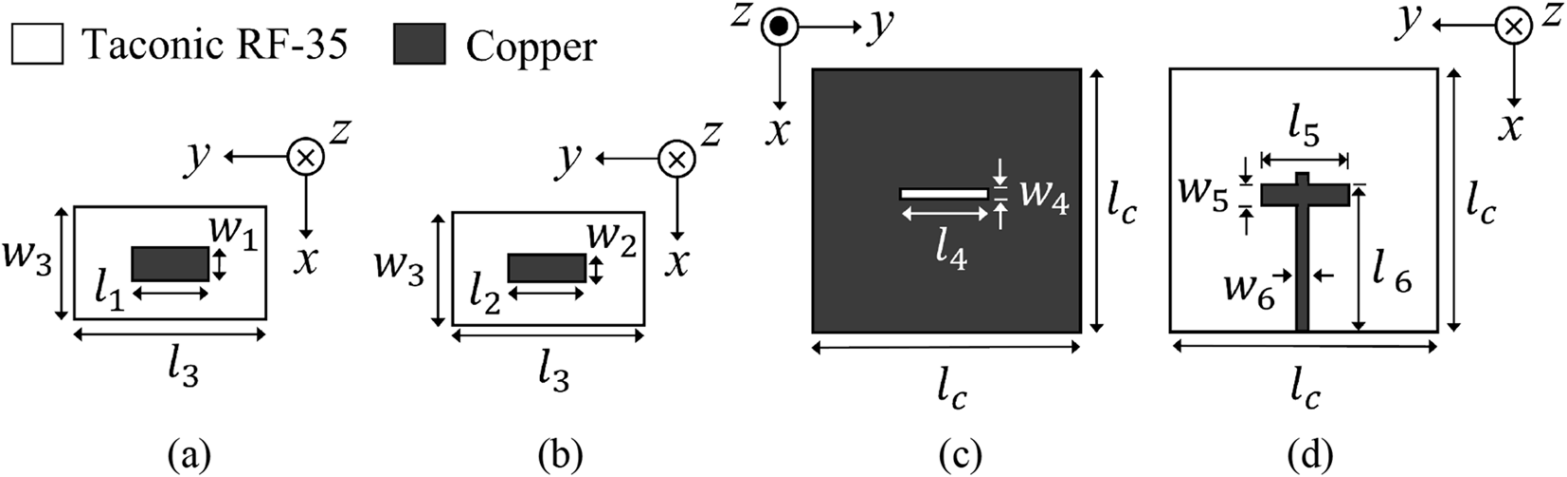
Exploded view of the source feeder: (**a**) 1^st^ patch layer, (**b**) 2^nd^ patch layer, (**c**) aperture slot layer, (**d**) transmission line layer with *l*_1_ = *l*_2_ = 18.5 mm, *l*_3_ = 37 mm, *l*_4_ = 12.5 mm, *l*_5_ = 13 mm, *l*_6_ = 132.92 mm, *l*_*c*_ = 260 mm, *w*_1_ = 14.53 mm, *w*_2_ = 10.5 mm, *w*_3_ = 29.06 mm, *w*_4_ = 1.85 mm, *w*_5_ = 5 mm, *w*_6_ = 3.46 mm [for 50 Ω impedance matching of a coaxial cable].

Figure 5 depicts the geometry of the source feeder, consisting of two metal layers (as shown in Fig. 1). For better impedance matching, the transmission line is terminated with a cross-shape stub optimized through simulation. The reflection coefficient and the 3D radiation power pattern of the feeder are shown in the Supplementary Figs. S1 and S2, respectively.

**Figure 6:**
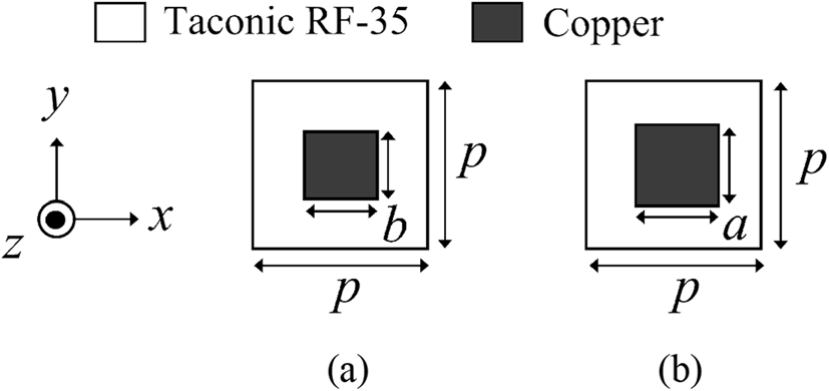
The geometry of a (**a**) PRS unit cell with *p* = 30 mm, *b* = 14 ~ 26 mm; and an (**b**) AMC unit cell with *a* = 10 ~ 28 mm.

The geometry of the PRS and AMC unit cells are also illustrated in Fig. 6. Each cell comprises a square metal patch on a Taconic RF-35 substrate. Only the bottom side of the AMC is fully covered with copper. The PRS and the AMC unit cells are designed to provide their reflection magnitudes in the ranges of $0.8 < |\Gamma_{n}^{{PRS{ }}} | < 0.95$ and $0.81 < |\Gamma_{n}^{{AMC{ }}} | < 1$, respectively.

**Figure 7:**
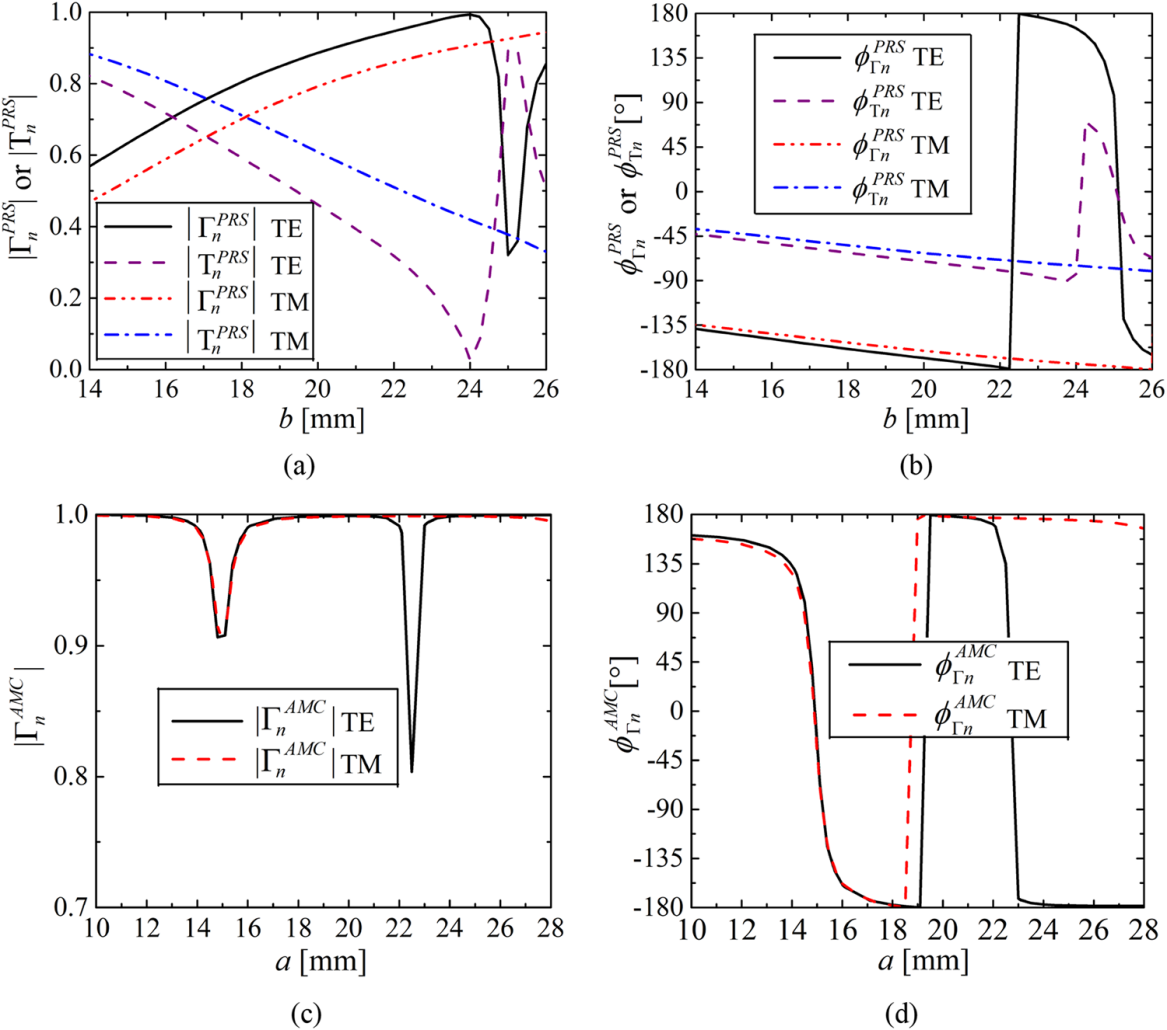
Reflection and transmission coefficients of both PRS and AMC cells (see Fig. 6) reveal distinct behaviors under TE and TM incidence that’s why we considered both incidences separately for the proposed TE and TM tapering at 5 GHz: (**a**) reflection $\left| {\Gamma_{n }^{{PRS{ }}} } \right|$ and transmission $\left| {{\text{T}}_{n}^{{PRS{ }}} } \right|$ magnitudes of the PRS, (**b**) reflection $\phi_{\Gamma n}^{PRS}$ and transmission $\phi_{\Gamma n}^{PRS}$ phases of the PRS, (**c**) reflection magnitude $\left| {\Gamma_{n }^{{AMC{ }}} } \right|$ of the AMC, and (**d**) reflection phase $\phi_{\Gamma n}^{AMC}$ of the AMC.

The reflection and transmission characteristics of the PRS and AMC unit cells are illustrated in Fig. 7. The cells exhibit distinct behaviors under different incidences (see Fig. 4), which demonstrates why we need to consider the TE and TM incidence separately in our antenna design. As we can see the reflection phases, the AMC cells provides significantly larger phase range (see Fig. 7d) than the PRS, which is favorable to construct the cavity resonance condition and advantageous for the highly-directive beamforming in the targeted direction.

The optimized magnitude and phase characteristics of the tapered PRS and the reflectarray are listed in Tables 1 and 2 under TE and TM incidences, respectively. Here, the high reflection magnitude of the PRS, $0.8\left\langle {|\Gamma_{n}^{PRS} } \right| < 0.95$, and the AMC, $0.81\left\langle {|\Gamma_{n}^{AMC} } \right| < 1$ are desirable for high SLS and maximizing realized gain.

**Table 1:** The reflection and transmission properties of the PRS superstrate and the reflectarray for TE incidence.

Values	*n* = 1	*n* = 2	*n* = 3	*n* = 4
$\left| {\Gamma_{n}^{PRS} } \right|$	0.95	0.9	0.85	0.8
$\left| {{\text{T}}_{n}^{PRS} } \right|$	0.3	0.43	0.52	0.59
$\phi_{\Gamma n}^{PRS}$	− 180°	− 173°	− 167°	− 161.5°
$\phi_{{{\text{T}}n}}^{PRS}$	− 80.5°	− 74°	− 68°	− 63°
$b$	26 mm	23.65 mm	21.68 mm	20.2 mm
$\phi_{\Gamma n}^{AMC}$	− 176°	177.5°	173°	N/A ^1^
$a$	17.55 mm	20.74 mm	26.43 mm	N/A

^1^N/A stands for not available.

**Table 2:** The reflection and transmission properties of the PRS superstrate and the reflectarray for TM incidence.

Values	*n* = 1	*n* = 2	*n* = 3	*n* = 4
$\left| {\Gamma_{n}^{PRS} } \right|$	0.95	0.9	0.85	0.8
$\left| {{\text{T}}_{n}^{PRS} } \right|$	0.3	0.43	0.52	0.59
$\phi_{\Gamma n}^{PRS}$	− 178°	− 170°	− 164°	− 158.5°
$\phi_{{{\text{T}}n}}^{PRS}$	− 80°	− 72.5°	− 66.5°	− 61.5°
$b$	22.14 mm	20.42 mm	19.04 mm	17.91 mm
$\phi_{\Gamma n}^{AMC}$	− 179°	174.5°	169.5°	N/A
$a$	18.62 mm	21.58 mm	22.05 mm	N/A

## Simulation and experiment results

### 1-D (TE or TM) tapering

**Figure 8:**
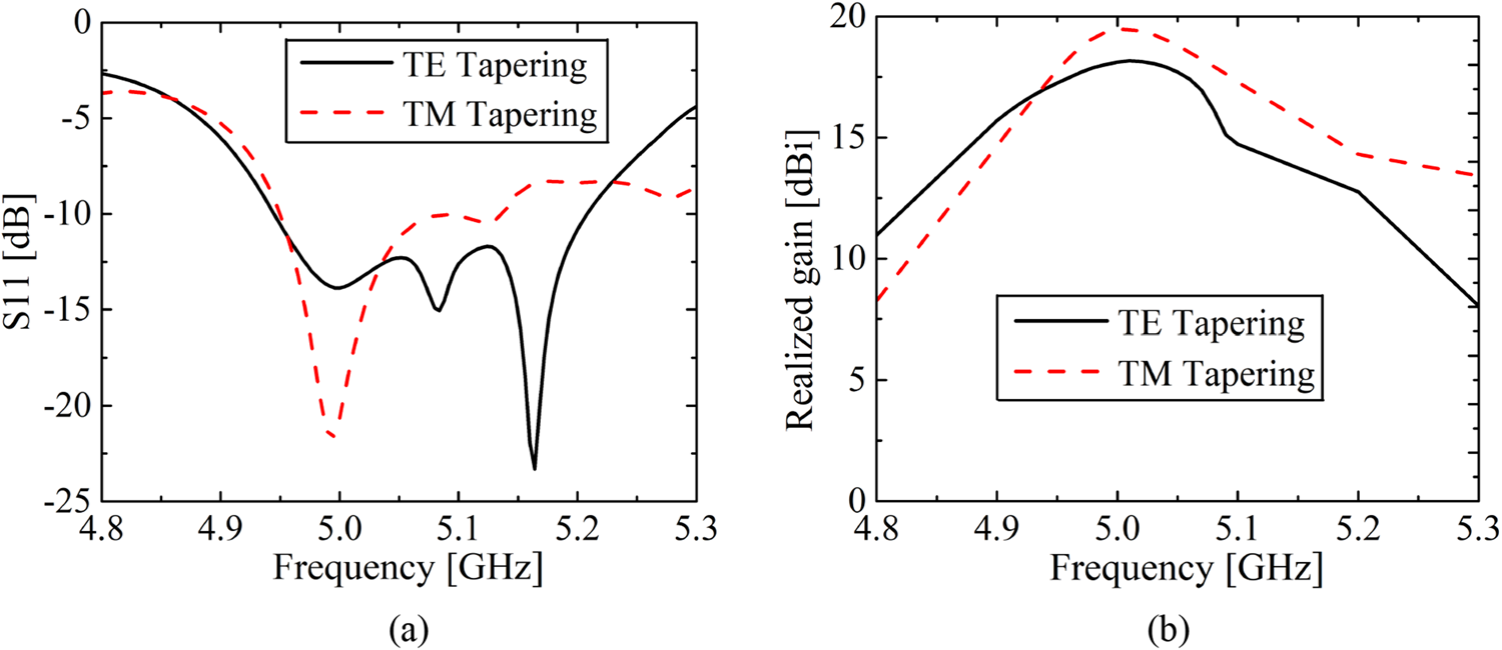
(**a**) Antenna reflection coefficient S11 and (**b**) peak realized gain of the 1-D TE tapering and 1-D TM tapering.

**Figure 9:**
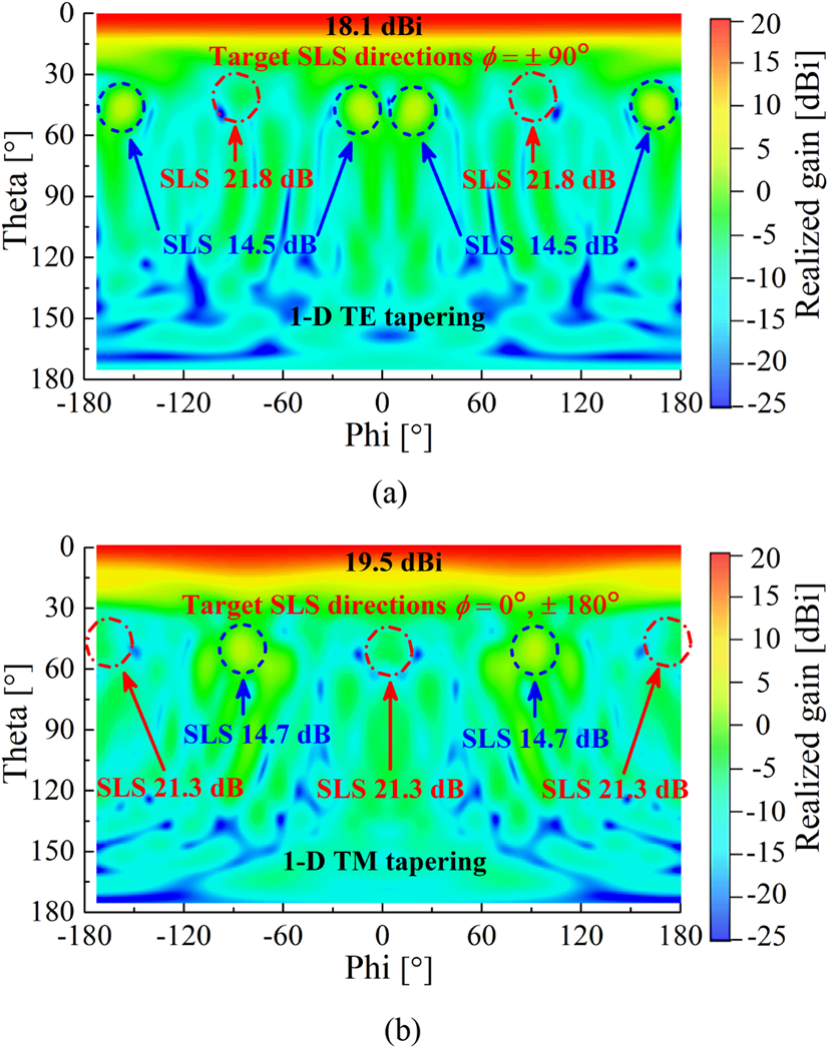
Radiation patterns (realized gain) after applying (**a**) 1-D TE tapering and (**b**) 1-D TM tapering to the *y*- (*ϕ* =  ± 90°) and *x*-axis (*ϕ* = 0° and 180°), respectively. Both are simulated at 5 GHz.

The impedance matching behavior and radiation patterns of the FPCA with 1-D (TE and TM) tapering are simulated and given in Figs. 8 and 9. The − 10 dB impedance bandwidth is well maintained around the design frequency of 5 GHz. Here, the TE tapering provides a broader bandwidth because AMC cells adjacent to the feeder behave differently for TE and TM design. As for the maximum realized gain, the TM tapering gives a higher gain around 5 GHz, as presented in Fig. 8b. For both cases, a gradual reduction of the gains at lower and higher frequencies is because of increasing deviation from FPC resonance and poor impedance matching.

The primary objective of the 1-D (TE or TM) tapering is to obtain high SLS along the corresponding tapering axis, with minimum reduction in an antenna gain. After applying the 1-D (TE or TM) tapering, radiation power patterns are illustrated in Fig. 9 to validate how much the SLS is enhanced. For the 1-D TE tapering applied along the *y*-axis, we expect high SLS along the axis (*ϕ* =  ± 90°), which is confirmed by high 21.8 dB SLS at *ϕ* =  ± 90° in Fig. 9a. Considering the relatively low SLS of 14.5 dB observed at *ϕ* =  ± 15° and ± 165° implies our tapering method is effective only around the tapering direction. Similarly, we can prove from Fig. 9b that the 1-D TM tapering also increases the SLS up to 21.3 dB in the directions (*ϕ* = 0° and 180°) where the tapering is applied. Again, the 14.7 dB SLS at *ϕ* =  ± 90° validates that tapering is effective only around the tapering-applied axis. The peak gains of the two tapering cases are 18.1 dBi (TE) and 19.5 dBi (TM), respectively. The 3D radiation patterns of the 1-D (TE or TM) tapering are illustrated in the Supplementary Figs. S3 and S4, respectively.

### 2-D (full TE-TM) tapering

**Figure 10:**
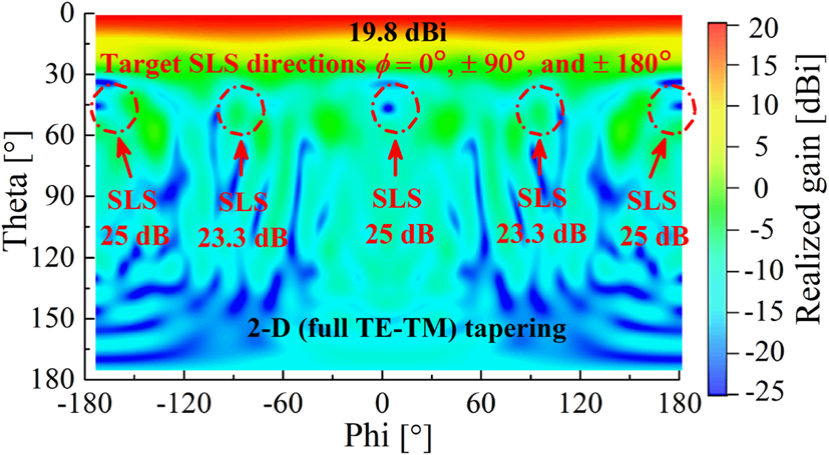
Radiation patterns (realized gain) after applying 2-D (full TE-TM) tapering at 5 GHz.

We move on to the 2-D (full TE-TM) tapering, which combines the 1-D TE and TM tapering, according to the way illustrated in Fig. 3b. Figure 10 shows a radiation pattern after applying the 2-D tapering. Here, we can find high SLS levels of 25 dB and 23.3 dB along the *x*- (*ϕ* = 0° and 180°, E-plane) and *y*- (*ϕ* =  ± 90°, H-plane) axis, respectively. Therefore, we can say that the proposed tapering method obviously helps suppress sidelobes even with no reflective side walls. It is also worth that the tapering increases the peak gain up to 19.8 dBi. The 3D radiation pattern of the 2-D (full TE-TM) tapering is illustrated in the Supplementary Fig. S5.

**Figure 11:**
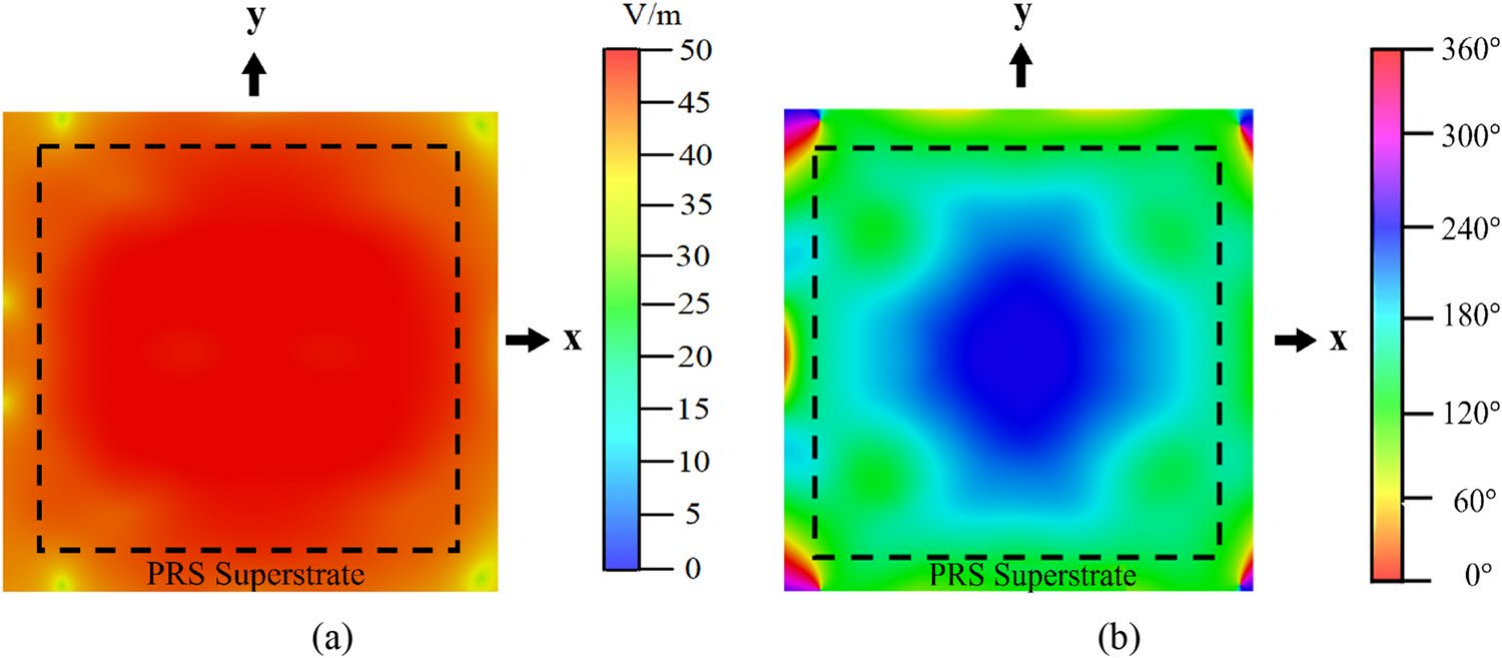
*E*_*x*_-field (**a**) magnitude and (**b**) phase distribution at 30 mm above the 2-D tapered PRS superstrate at 5 GHz.

To check the validity of our design in terms of 3-D field distribution, we pick up the magnitude and the phase distributions of the dominant *E*_*x*_-field 30 mm above the PRS, which is given in Fig. 11. The square with dotted lines is a footprint of the PRS. Both distributions change little around the *x*- and *y*-axis, which implies the antenna satisfies the FPC resonance condition very well. However, they vary more significantly in the diagonal directions, so there is still room for further SLS in the diagonal cell design, which is a future research topic.

**Figure 12:**
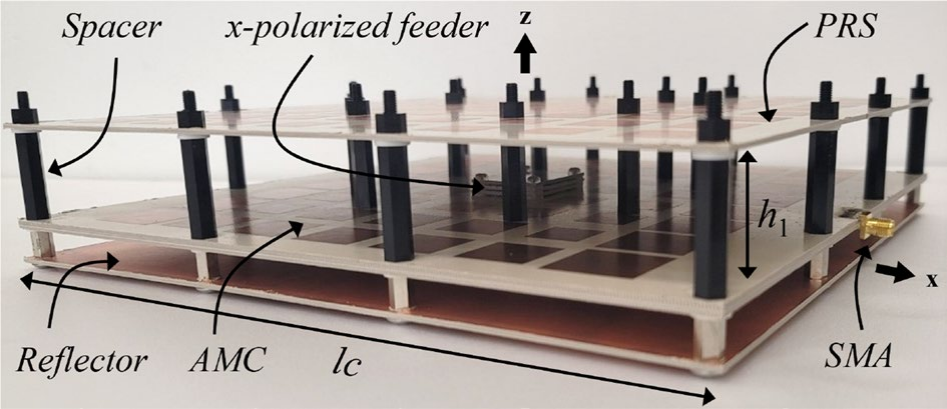
Fabricated FPCA with 20 plastic M4 spacers.

The fabricated antenna is shown in Fig. 12, which consists of 64 PRS and 54 AMC cells measuring 260 mm (4.3 *λ*_0_) × 260 mm (4.3 *λ*_0_) × 31.6 mm (0.52 *λ*_0_). The FPCA is very sensitive to cavity height, so we used 20 plastic M4 spacers to keep the PRS as flat as possible.

**Figure 13:**
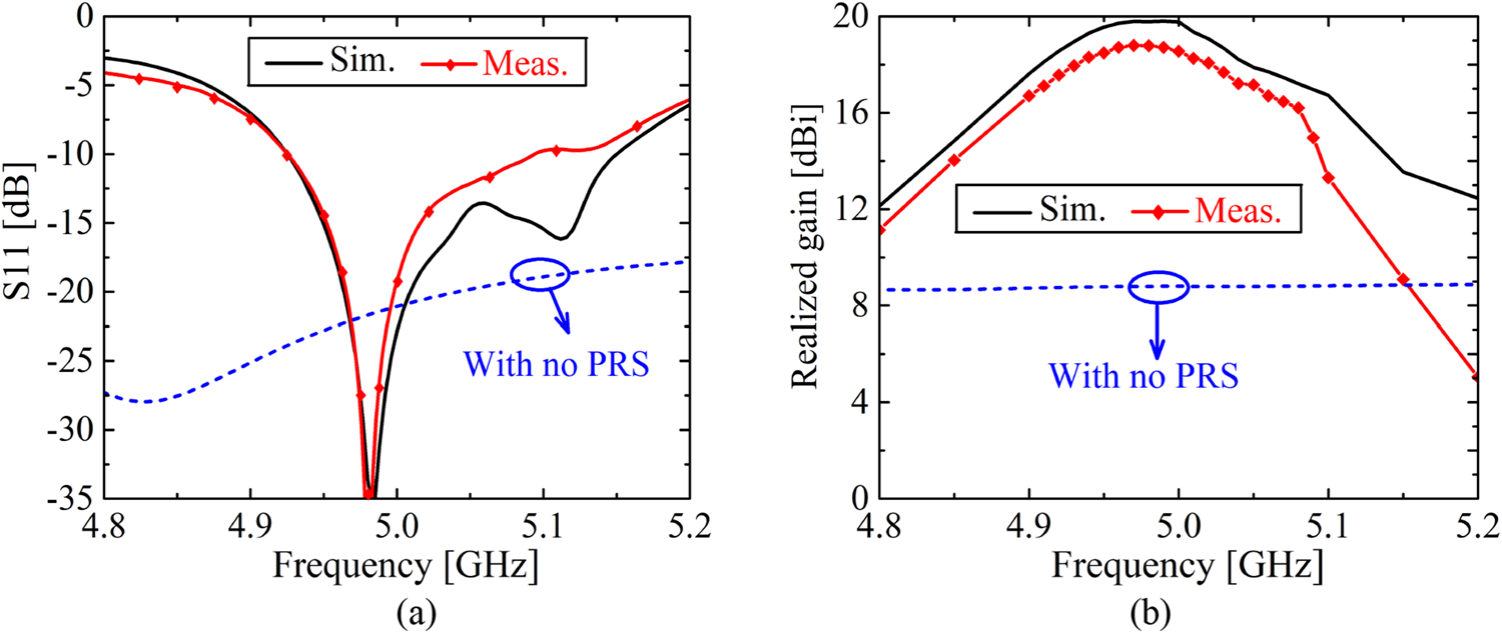
(**a**) Antenna reflection coefficient S11, and (**b**) peak realized gain of the FPCA with and without the 2-D (full TE-TM) tapering applied PRS.

The impedance matching and the peak realized gain behaviors of the FPCA with and without 2-D tapering applied PRS are given in Fig. 13. The measured − 10 dB impedance bandwidth is about 187 MHz, corresponding to 3.7% fractional bandwidth, which follows the simulation relatively well. The impedance measurement setup is shown in the Supplementary Fig. S6. As for the maximum realized gain, the measured peak gain is 18.8 dBi, which is 1 dB lower than the simulation. The deviation between the gains is greater at higher frequencies because the PRS is not completely flat, which is a common problem resulting from a chemical etching process. In this work, we used 20 spacers to keep the PRS as flat as possible, but in the end, we could not make it perfectly flat. That explains the large gain deviation in higher frequencies.

**Figure 14:**
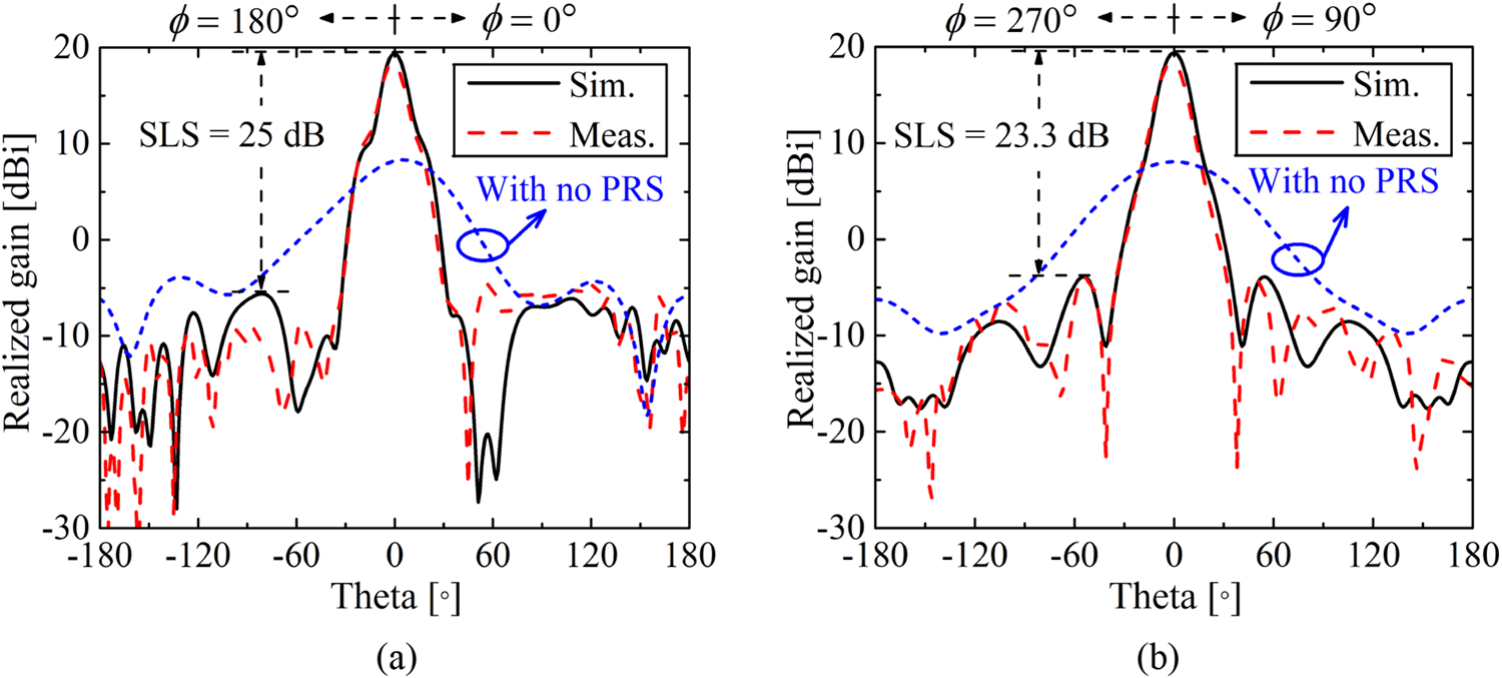
Radiation patterns (realized gain) along (**a**) *x*-axis (*ϕ* = 0° & 180°, E-plane) and (**b**) *y*-axis (*ϕ* =  ± 90°, H-plane) of the 2-D (full TE-TM) tapering of the FPCA with and without PRS at the *f*_0_ = 5 GHz. Here, sidelobes are well suppressed below 23.3 dB on both E- and H-planes thanks to a proposed 2-D tapering method.

The radiation patterns of the FPCA with and without 2-D tapering applied PRS are also given in Fig. 14. The measured radiation pattern of the FPCA is done in an echoic chamber, which is depicted in the Supplementary Figs. S7 and S8, respectively. We can see that sidelobes on both the E- (*ϕ* = 0° and 180°, *x*-axis) and H-planes (*ϕ* = 90° and 270°, *y*-axis) are suppressed well below 23.3 dB thanks to the proposed 2-D tapering method. Half power beam widths are 14.4° and 16.4° on the E- and H-planes, respectively. The sidelobe suppression along *ϕ* = 45° and 225° is depicted in Supplementary Fig. S9.

Our antenna suppresses side lobes the best with a reasonably high antenna gain and aperture efficiency. Some important antenna performance according to the applied tapering methods is summarized in Table 3. Our work is also compared with recently published related works in Table 4.

**Table 3:** Antenna performance variation according to the tapering methods.

Antenna performance	Tapering method			
	Simulation			Measured
	1-D (TE)	1-D (TM)	2-D (TE-TM)	2-D (TE-TM)
Impedance bandwidth ^1^	263 MHz	187 MHz	236 MHz	186 MHz
Fractional bandwidth	5.1 %	3.7 %	4.5 %	3.7 %
Realized gain	18.1 dBi	19.5 dBi	19.8 dBi	18.8 dBi
Radiation efficiency	90 %	90 %	91 %	91 %
Total efficiency	87 %	89 %	90 %	90 %
Aperture efficiency	28 %	38 %	41 %	35 %
SLS (*x*-axis, *ϕ* = 0° & 180°)	14.5 dB	21.3 dB	25 dB	23.5 dB
SLS (*y*-axis, *ϕ* = ± 90°)	21.8 dB	14.7 dB	23.3 dB	23.3 dB

^1^Measured at S11 =  − 10 dB.

**Table 4:** Antenna performance comparison with previous FPCAs.

Refs.	*f*_0_ (GHz)	FBW ^1^ (%)	No. of PRS layers	PRS thickness (mm)	Aperture area (*λ*_0_^2^)	Antenna height (*λ*_0_)	Realized gain (dBi)	Aperture efficiency (%)	SLS (dB) *x*-axis, *ϕ* = 0° and 180°	SLS (dB) *y*-axis, *ϕ* = ± 90°
^23^	61.4	14.8	1	1.27	10.09	0.47	11.00	9.9	4.5	15.4
^24^	13.5	14.4	1	1.575	10.24	0.60	17.90	47.9	10.0	11.0
^25^	7.0	N/A ^2^	1	5.08	3.14	0.50	14.03	64.0	14.9	14.9
^26^	10.0	30.0	3	23.30	4.00	1.34	17.40	27.3	9.0	12.0
^27^	5.36	3.5	1	1.578	4.29	0.10	9.12	15.14	9.0	10.0
^28^	5.8	1.4	2	7.00	11.76	0.52	20.78	80.9	20.0	20.0
^29^	5.8	4.7	2	17.00	9.62	0.68	19.40	72.0	17.0	17.0
This work	5.0	5.1	1	1.52	18.75	0.52	19.80	40.5	25.0	23.3

^1^Fractional bandwidth, ^2^N/A stand for not available.

## Conclusion

In this work, we have proposed a novel FPCA to improve SLS property while maintaining a high antenna gain. We have applied the 1-D and 2-D tapering to the reflection magnitude of the PRS to suppress the sidelobes. Moreover, we have used additional AMC cells along with the PRS to make the FPC satisfy Trentini’s resonance, which helps maintain a reasonably high gain.

Accordingly, our FPCA has attained more than 23 dB high SLS with a peak realized gain of 19.8 dBi.

The proposed FPCA has a simple configuration but simultaneously provides a relatively high gain and SLS. Therefore, we expect our antenna to find many potential applications requiring a high gain and SLS, such as 5G communications, radars, satellite systems, and urban air mobility (UAM).

### Supplementary Information

Supplementary Information.

## Data Availability

All data required to evaluate the findings of this work is available in the presented paper. Additional data related to this work may be requested from the corresponding author.
