# Supplementary material for: Tapered high-gain Fabry–Perot cavity antenna with high sidelobe suppression for 5G industry
Source: Sci Rep. 2023 Sep 21;13:15744. doi: 10.1038/s41598-023-42716-8 (PMC10514039; doi:10.1038/s41598-023-42716-8)
Supplement: Supplementary file 1 — Supplementary Information. [file 41598_2023_42716_MOESM1_ESM.docx]

Supplementary Information


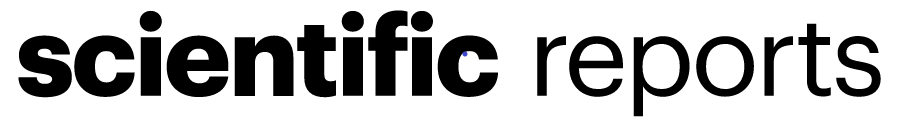


Tapered High-Gain Fabry-Perot Cavity Antenna with High Sidelobe Suppression for 5G Industry

1. **The rigorous derivation of the overall phase delay within the cavity**

In the paper, (1) is Trentini’s beamforming condition, which is exploited by Von Trentini in [30]. An equation (2) is the overall phase delay of each ray (as shown in Fig. 1) to construct the plane wavefront for high realized gain in the targeted direction, which is derived from the ray-tracing method [30]. Its rigorous derivation is the following:

The phase delay 𝜑_1_ of the first ray from the feeder to the wavefront is

|  | (1) |
| --- | --- |

The phase delay 𝜑_2_ of the second ray from the feeder to the wavefront is

|  | (2) |
| --- | --- |

The phase delay 𝜑_3_ of the third ray from the feeder to the wavefront is

|  | (3) |
| --- | --- |

Similarly, the phase delay 𝜑_n_ of the *n*^th^ ray from the feeder to the wavefront is

|  | (4) |
| --- | --- |

But for the proposed tapered PRS, both its reflection and transmission phases play a key role in producing high gain. As we know, it is quite challenging to control the reflection and transmission phase of the PRS unit cell, independently. In other words, it is hard to maintain strong resonance in the cavity without being affected by transmission behavior. Moreover, a single-layer PRS unit cell cannot provide wide enough phase coverage to control the cavity phase response. To overcome these issues, we introduce an additional AMC unit cell that provides a desirable wide-enough reflection phase to form a wavefront in the target direction. Therefore, the reflection phase of the AMC unit cell will be derived for the planner wavefront.

Respecting the ray theory, all phase delays (𝜑_1_, 𝜑_2_, 𝜑_3_,…., 𝜑_n)_ are equal at the wavefront. To calculate the reflection phase of the first AMC unit cell, phase delay 𝜑_1_ will be equal to phase delay 𝜑_2_ regarding ray theory.

| ** | (5) |
| --- | --- |
| ** | (6) |

For the reflection phase of the second AMC unit cell, phase delay 𝜑_2_ will be equal to phase delay 𝜑_3_.

| ** | (7) |
| --- | --- |
| ** | (8) |

Similarly, the reflection phase of the *n*^th^ AMC unit cell will be

|  | (9) |
| --- | --- |

Here, 𝛳*_t_* is the targeted direction of the radiation pattern of the main beam (e.g., 𝛳*_t_* = 0° for beam pattern towards the broadside direction).

1. **PRS and AMC unit cells deployment for 1-D TE or TM tapering**

In Figure 3(a), the PRS and AMC (yellow rows) are deployed for 1-D TE tapering along *y*-axis (𝜙 = 90° and 270°) for high sidelobe suppression. Only one row at center AMC (blue) is deployed along the *x*-axis to attain the sidelobe suppression. Using (6), reflection phase ($\phi_{Г1}^{AMC}$) of AMC is calculated regarding the TM reflection characteristics of the PRS unit cell ($\left| Г_{1}^{PRS} \right|$, $\phi_{Г1}^{PRS}$ and $\phi_{T1}^{PRS}$). As a result, we can see 14.5 dB sidelobe suppression along the *x*-axis (𝜙 = 0° and 180°) in Figure 9a for 1-D TE tapering. Vice versa, we can see 14.7 dB sidelobe suppression along the y-axis (𝜙 = 90° and 270°) in Figure 9b for 1-D TM tapering.

1. **The results of the feeder**

The reflection coefficient and 3D radiation pattern of the feeder (aperture-coupled stacked microstrip patch antenna) are shown in Figures S1 and S2, respectively.


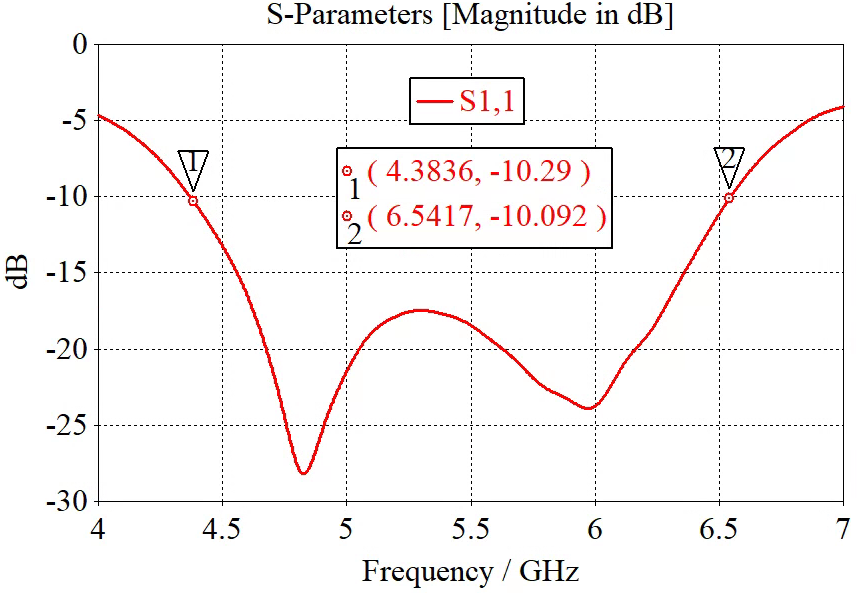


**Figure S1.** The reflection coefficient of the feeder.


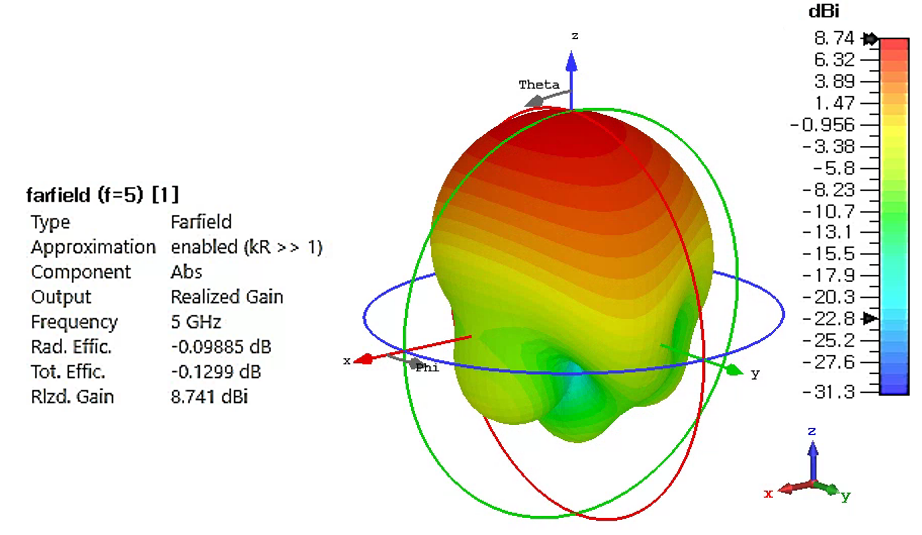


**Figure S2.** The 3D radiation pattern of the feeder.

1. **The results of the 1-D (TE or TM) Tapering**

The 3D radiation patterns of the 1-D (TE or TM) tapering are shown in Figures S3 and S4, respectively.


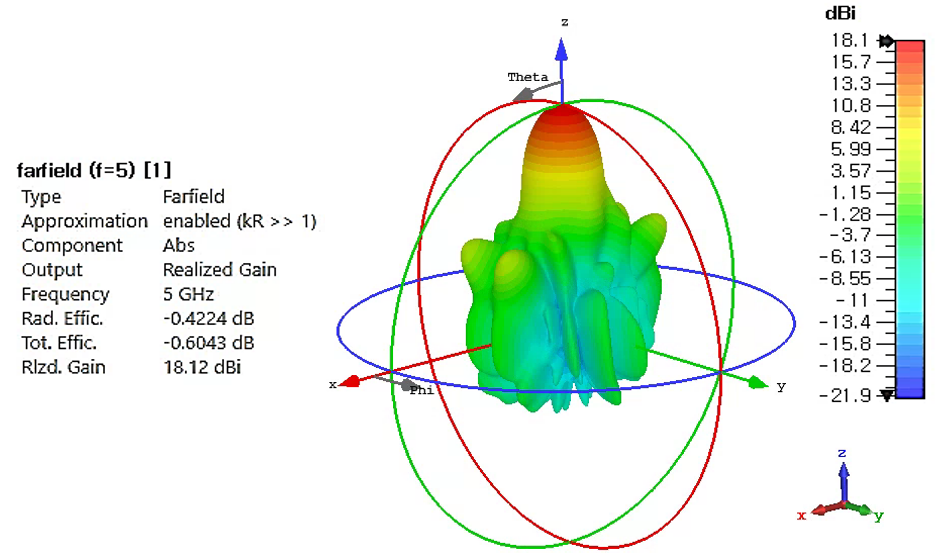


**Figure S3.** The 3D radiation pattern of the 1-D TE tapering.


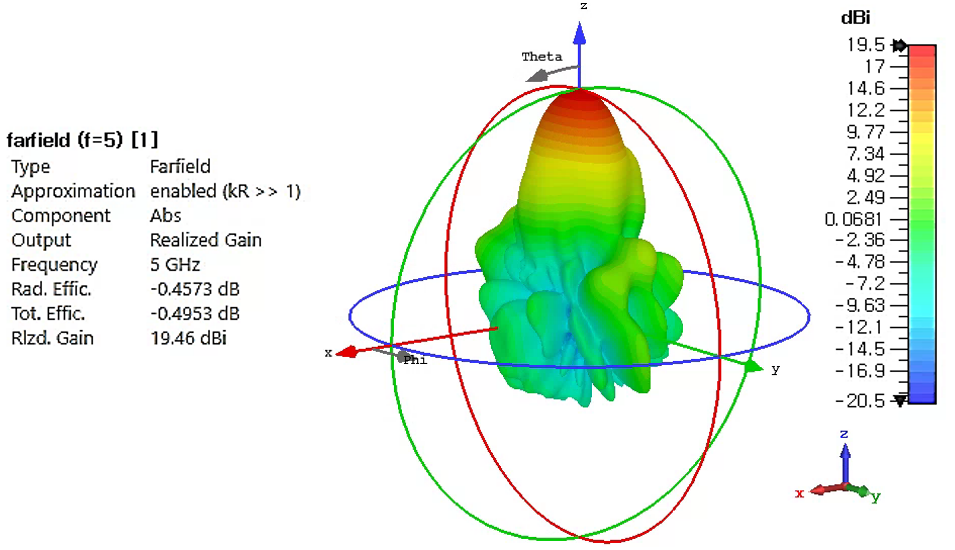


**Figure S4.** The 3D radiation pattern of the 1-D TM tapering.

1. **The result of the 2-D (fully TE-TM) Tapering**

The 3D radiation pattern of the 2-D (fully TE-TM) tapering is shown in Figure S5.


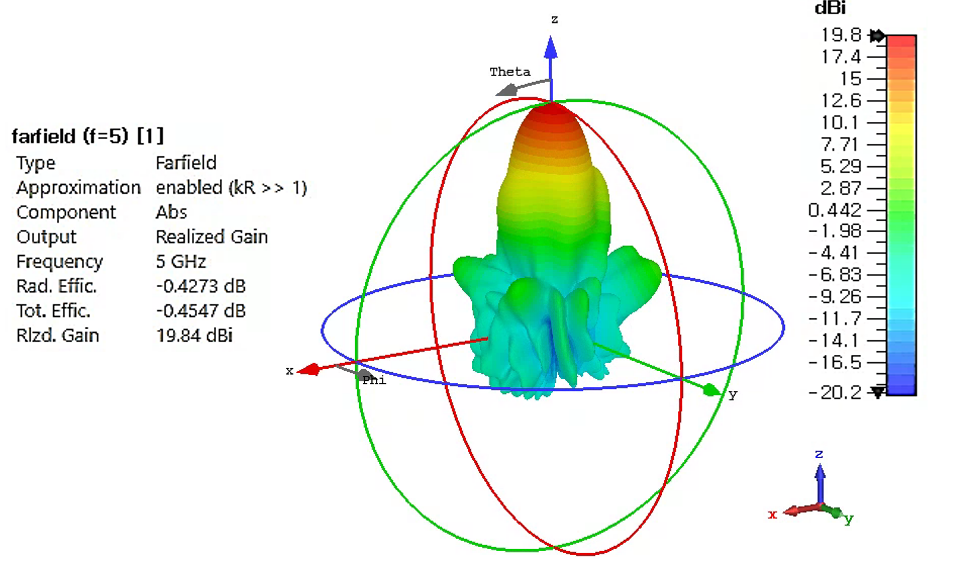


**Figure S5.** The 3D radiation pattern of the 2-D (fully TE-TM) tapering.

1. **The impedance measurement setup of the 2-D (fully TE-TM) Tapering**

The impedance measurements setup of the 2-D (fully TE-TM) tapering is depicted in Figure S6, which is performed on a 40 GHz vector network analyzer (Anritsu VNA; MS46122A) using the short-open-load (SOL) —Anritsu calibration kit; TOSLKF50A-40, type K(f), 50 Ω— standardized calibration approach.

*
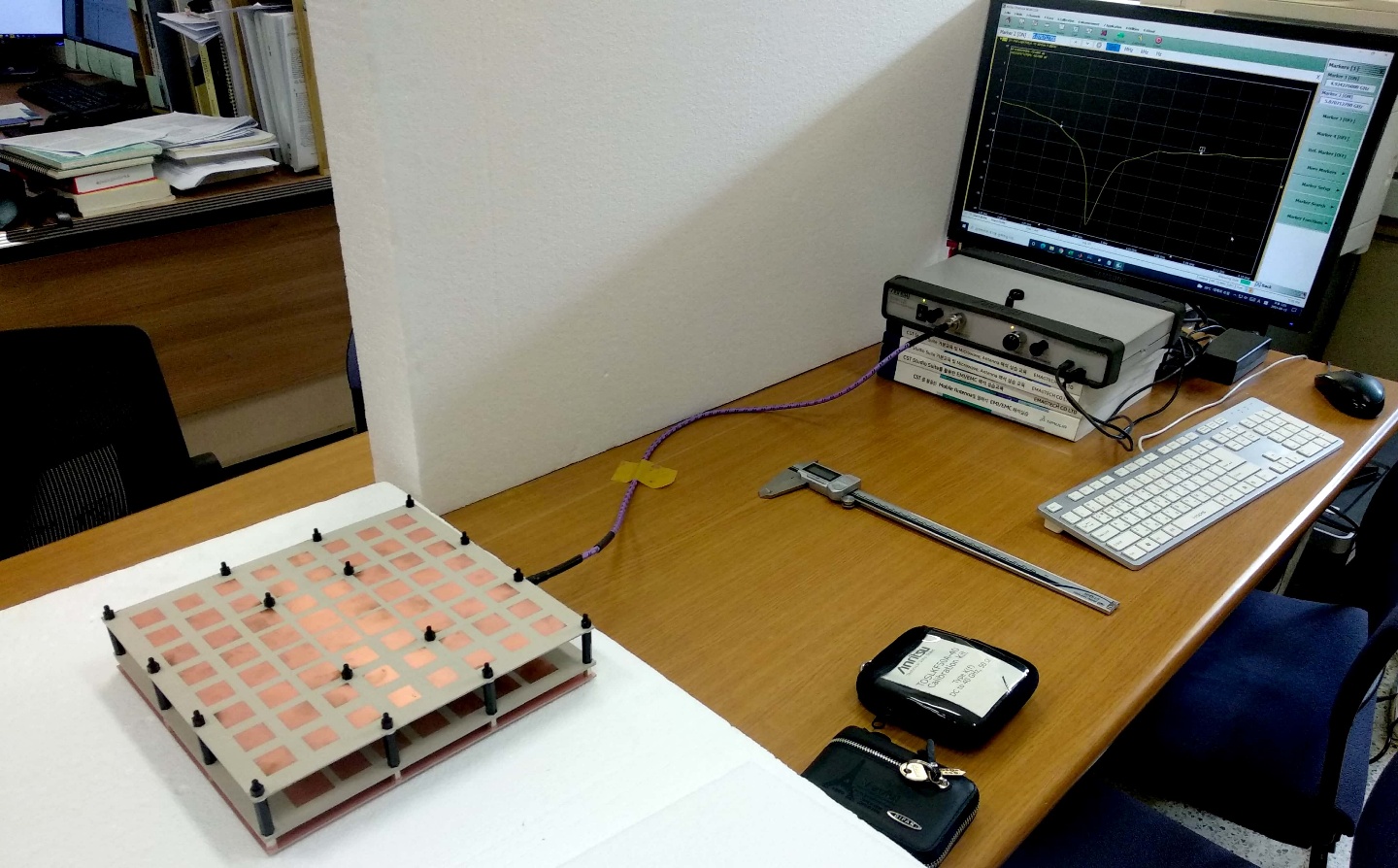
*

**Figure S6.** The impedance measurements setup of the 2-D (fully TE-TM) tapering.

1. **An echoic chamber measurement setup of the 2-D (fully TE-TM) Tapering**

*
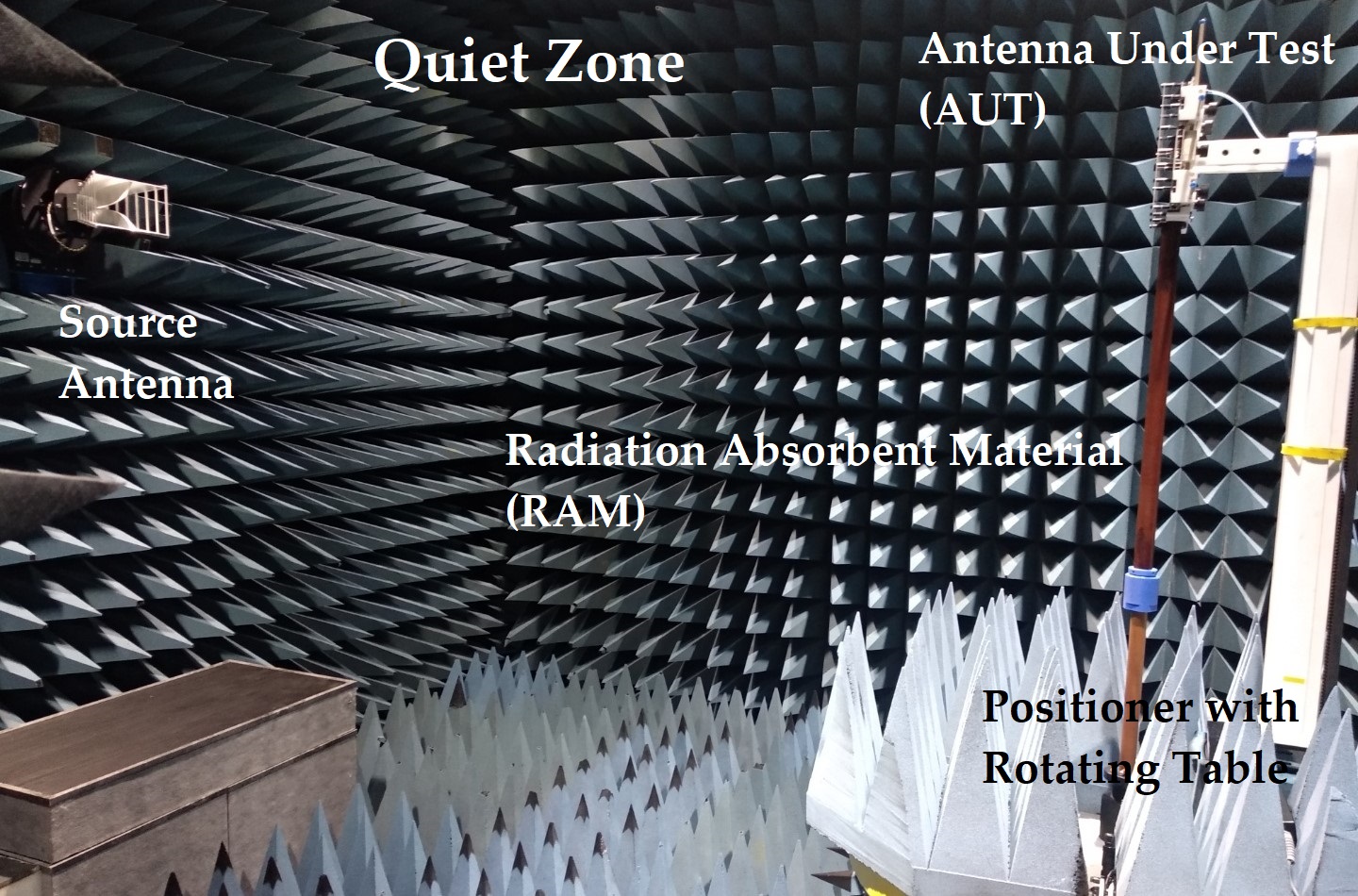
*

**Figure S7.** Anechoic chamber measurements setup of the 2-D (fully TE-TM) tapering.


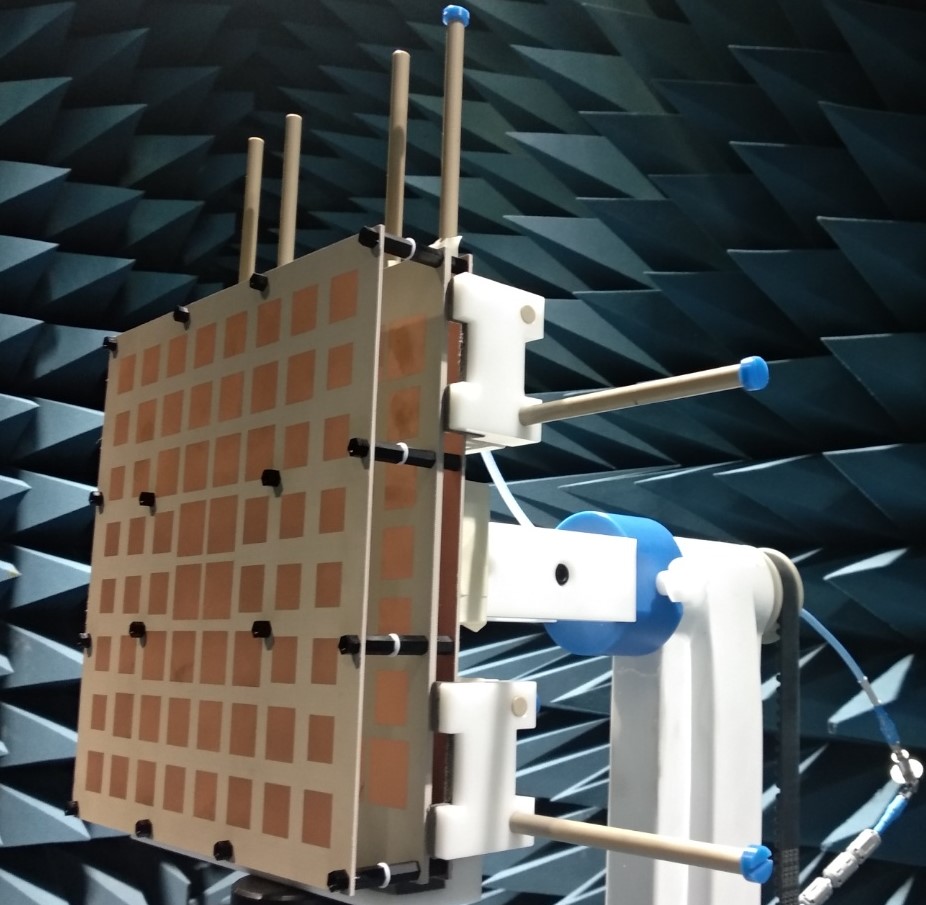


**Figure S8.** The FPC antenna under test in the chamber measurements setup of the 2-D (fully TE-TM) tapering.

1. **The radiation pattern (realized gain) of the 2-D (fully TE-TM) Tapering along 𝜙 = 45° and 225°**

Regarding the 2D (full TE and TM) tapering case, the sidelobe level along 𝜙 = 45° and 225° is shown in the following Figure S9. Here we can see that a 22.6 dB sidelobe level is observed, which is also highly suppressed just like E- (𝜙 = 0° and 180°) and H-planes (𝜙 = 90° and 270°). It implies that our proposed strategy of tapering is an effective feature for high sidelobe suppression.


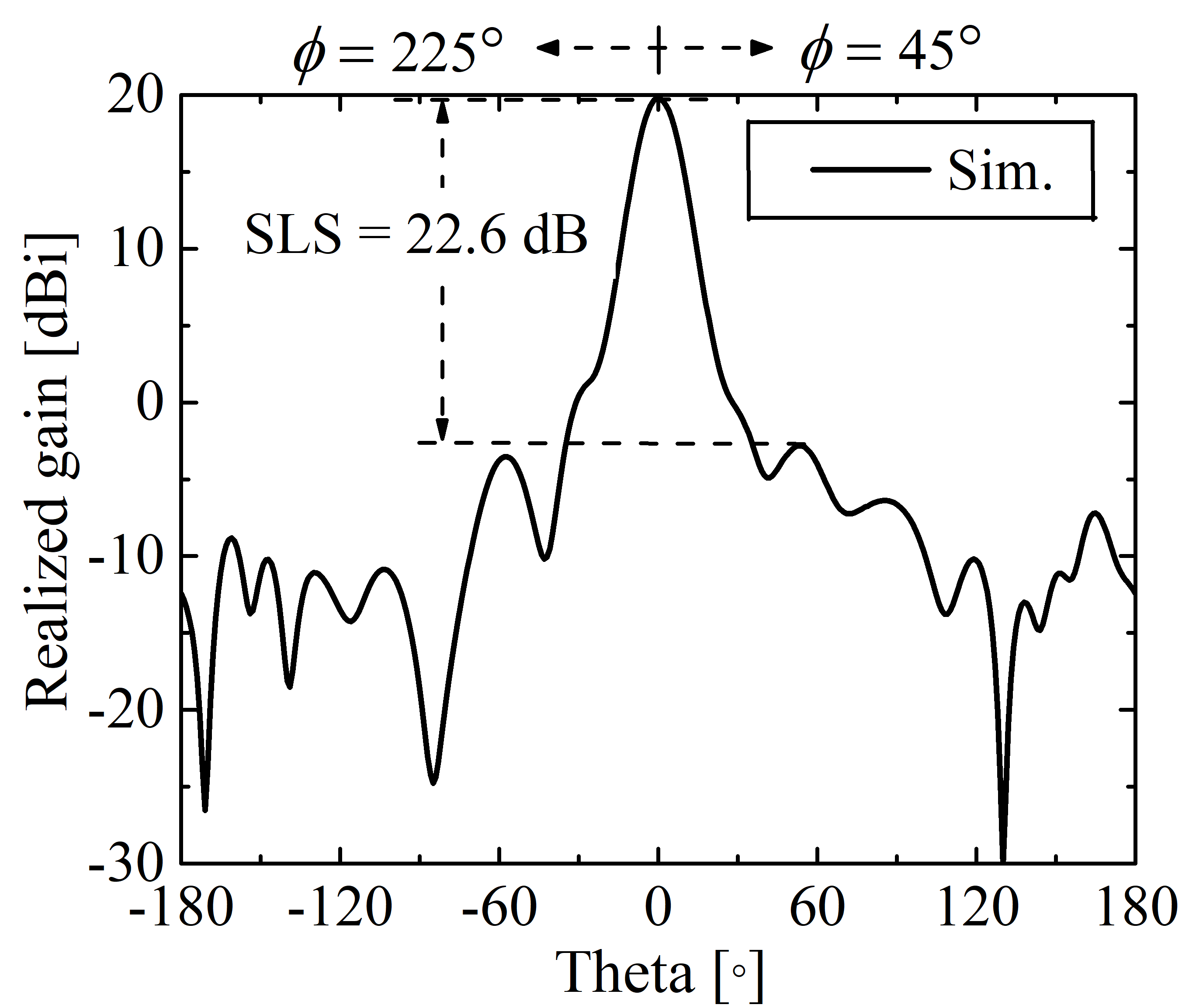


**Figure S9.** The 2D radiation pattern along *ϕ* = 45° & 225° of the 2-D (full TE-TM) tapering of the FPCA at the *f*_0_ = 5GHz. Here, 22.6 dB sidelobes are suppressed.
